# Renal Effects of Fetal Reprogramming With Pentaerythritol Tetranitrate in Spontaneously Hypertensive Rats

**DOI:** 10.3389/fphar.2020.00454

**Published:** 2020-04-29

**Authors:** Andy W. C. Man, Min Chen, Zhixiong Wu, Gisela Reifenberg, Andreas Daiber, Thomas Münzel, Ning Xia, Huige Li

**Affiliations:** ^1^Department of Pharmacology, Johannes Gutenberg University Medical Center, Mainz, Germany; ^2^Department of Anaesthesiology, Institute of Anaesthesiology and Critical Care Medicine, Union Hospital, Tongji Medical College, Huazhong University of Science and Technology, Wuhan, China; ^3^Center for Cardiology, Cardiology I - Laboratory of Molecular Cardiology, Johannes Gutenberg University Medical Center, Mainz, Germany; ^4^German Center for Cardiovascular Research (DZHK), Partner Site Rhine-Main, Mainz, Germany

**Keywords:** fetal programming, epigenetics, spontaneously hypertensive rats, pentaerythritol tetranitrate, kidney fibrosis

## Abstract

**Aims:**

Current antihypertensive therapies cannot cure hypertension and a life-long medication is necessary. Maternal treatment may represent a promising strategy for hypertension treatment. We have previously shown that maternal treatment of spontaneously hypertensive rats (SHR) with pentaerythritol tetranitrate (PETN) leads to a persistent blood pressure reduction in the female offspring. The underlying mechanisms include improved endothelial function resulting from long-lasting epigenetic changes. In the present study, we address the renal effects of maternal PETN treatment.

**Methods and Results:**

F0 parental SHR were fed with either normal chow or PETN-containing (1 g/kg) chow ad libitum from the time point of mating to the end of lactation period. The F1 offspring received normal chow without PETN from the time point of weaning (at the age of 3 weeks). At the age of 16 weeks, female PETN offspring showed lower blood pressure than the control. No difference was observed in male offspring. All following experiments were performed with kidneys from 16-week-old female offspring. Maternal PETN treatment reduced the mRNA and protein expression of angiotensin-converting enzyme (ACE) and basic fibroblast growth factor (FGF2), resulting from epigenetic modifications found at the proximal promoter regions. The expression levels of mineralocorticoid receptor (MR) and factors in the MR signalling pathway (Rac1 and Sgk1) were also reduced by PETN. Major profibrotic cytokines, including Wnt4, TNF-alpha, TGF-beta, and MMP9, were downregulated by PETN, which was associated with reduced collagen deposition and glomerulus sclerosis in the kidney of PETN offspring. In addition, PETN treatment also decreased the markers of inflammation and immune cell infiltration in the kidneys.

**Conclusions:**

PETN maternal treatment leads to epigenetic changes in the kidney of female SHR offspring. The reduced renal inflammation, alleviated kidney fibrosis, and decreased MR signalling are potential mechanisms contributing to the observed blood pressure-lowering effect.

## Introduction

Hypertension is the most common chronic disease in human. Hypertension is also the major risk factor for various renal-cardiovascular complications including heart failure, stroke, and kidney diseases (Lawes et al., 2001; Crowley and Coffman, 2014), which lead to substantial morbidity and mortality (Trial, 1967). Current antihypertensive treatments involving life-long administration of multiple drugs, cannot completely cure hypertension and yet a significant number of patients show resistance toward antihypertensive medications (Van Jaarsveld et al., 2001; Gu et al., 2012). Therefore, there is a need for novel types of antihypertensive therapy. Epigenetic studies of hypertension hold great promise of providing novel insights into the mechanisms underlying hypertension (Liang et al., 2013), which may be helpful in developing new therapeutic strategies.

The sensitivity of the epigenome to the environment decreases during life as growth slows; pregnancy and lactation periods are both within the time window of high susceptibility of the epigenome (Gluckman et al., 2009). Epigenetic modifications can be induced by treatment during pregnancy and lactation periods, even with compounds that are not direct inhibitors or activators of the epigenetic machinery enzymes (i.e., DNMT, HDAC, etc.). Previous studies have shown a persistent blood pressure reduction in spontaneously hypertensive rats (SHR) offspring from mother SHR that were treated during the last 2 weeks of gestation and until 4 or 8 weeks of age with either a combination of L-arginine plus vitamins C and E and taurine (Racasan et al., 2004), with the nitric oxide (NO) donor molsidomine (Racasan et al., 2005) or with the L-arginine precursor L-citrulline (Koeners et al., 2007). These effects may very likely involve epigenetic mechanisms, although epigenetic changes were not analyzed in those studies.

In a recent study, we treated the parent F0 SHR from the time point of mating to weaning of the F1 offspring with pentaerythritol tetranitrate (PETN). PETN treatment had no effect on the blood pressure of the F0 animals, or of the male offspring. Interestingly, treatment of F0 parent SHR during the pregnancy and lactation periods led to a clear blood pressure reduction in the female offspring. Because the F1 rats had no contact with PETN from the time point of weaning, the blood pressure reduction in the female offspring represents a long-lasting effect that persisted over a time period of at least 8 months (the experiment was terminated at the age of 8 months) (Wu et al., 2015). As molecular mechanisms, we have found that maternal PETN treatment causes to epigenetic changes leading to an improvement of endothelial function. SHR are a rat model of genetic hypertension. These results indicate that maternal treatment may represent an effective epigenetic therapy, even for diseases that are caused mainly by genetic factors.

In our previous study, we focused on the effect of maternal PETN treatment on blood vessels. In addition to peripheral vascular resistance, kidney function is another key factor regulating blood pressure (Cowley and Roman, 1996). The relationship between renal sodium balance, intravascular fluid volume homeostasis, and hypertension was initially described by Guyton et al. (1972). Angiotensin-converting enzyme (ACE) is implicated in the progression of kidney dysfunction and structural damages to chronic kidney diseases and associated hypertension (Anderson et al., 1985; Rosenberg et al., 1994). In human genetic studies, all identified genetic mutations associated with hypertension involve renal-proteins (Lifton et al., 2001). Therefore, kidney function is critical for blood pressure control. Kidney dysfunction includes the pathologies of inflammation and formation of fibrosis. At the stage of prehypertension, renal inflammation is already provoked by augmented macrophage infiltration, which also cause vasoconstriction and promote sodium and water reabsorption (Harrison et al., 2011). The presence of early inflammatory events in the kidney may be critical by introducing irreversible processes that can lead toward organ damage. Prolonged inflammation in kidney stimulates the excess collagen deposition and leads to the formation of renal fibrosis which have also been demonstrated to be involved in the development of hypertension (Johnson et al., 1992). These kidney pathologies are associated with basic fibroblast growth factor (FGF2), an important cytokine which facilitates renal matrix formation (Strutz et al., 2001; Mattison et al., 2012; Bozic et al., 2018).

SHR is an essential hypertension animal model (Yamori, 1977). In the SHR, aging is a significant factor affecting vascular endothelial dysfunction. Endothelial dysfunction only exists in SHRs older than 6 months; but not in younger SHRs (Bernatova et al., 2009). However, a significant increase in inflammatory markers in the kidney was already demonstrated at earlier stage in SHR (Heijnen et al., 2014). Therefore, we want to investigate the effects of maternal PETN treatment on the kidney of the offspring and the experiments were conducted at the age of 4 months.

## Method

### Animal Model

SHR were obtained from Charles River Laboratories (Sulzfeld, Germany). PETN (18% PETN with 82% D-lactose monohydrate) was provided by Actavis Deutschland GmbH (now PUREN Pharma GmbH & Co. KG, Munich, Germany). PETN-lactose (5.5 g/kg; ≈ 1 g/kg PETN) was mixed into normal chow diet (ssniff GmbH, Soest, Germany) resulting in a PETN dose of approximately 50 mg/kg/day. F0 parental SHR were fed with either normal chow (control) or PETN-containing chow ad libitum from the time point of mating (at the age of three month) to the end of lactation period. The F1 offspring rats from all groups received normal chow without PETN after weaning (at the age of 3 weeks). Totally, five control breading pairs and five PETN breading pairs were used. Twenty-five female offspring from the control breading pairs and 26 female offspring from the PETN breading pairs were included in the study. All the experiments performed involve offspring from at least three different litters. The animal experiment was approved by the responsible regulatory authority (Landesuntersuchungsamt Rheinland-Pfalz; 23 177-07/G 11-1-015) and was conducted in accordance with the German animal protection law and the National Institutes of Health (NIH) Guide for the Care and Use of Laboratory Animals.

### Blood Pressure Measurement

Systolic blood pressure, diastolic blood pressure, and mean blood pressure were measured noninvasively in conscious animals by using a computerized system (CODA Monitor, Kent Scientific) with a volume-pressure recording sensor and an occlusion tail-cuff. Rats were placed in individual holders. The occlusion cuff and the volume-pressure recording cuff were placed close to the base of the tail. After an adaptation period of 30 min on a 37°C warm pad, and five preliminary measurement was performed before actual measurement. Results are presented as the mean of at least 15 recordings on each occasion taken. The measurements were performed at the same time of a day by the same investigator as done in our previous studies (Wu et al., 2015).

### Gene Expression Studies by Quantitative PCR

Total RNA of the rat kidney was isolated using peqGOLD TriFast™ (PEQLAB) and cDNA was reverse transcripted using High Capacity cDNA Reverse Transcription Kit (Applied Biosystems) according to manufacturer’s instructions. Quantitative PCR (qPCR) were performed using SYBR Green JumpStart™ *Taq* Ready-Mix™ (Sigma-Aldrich) on an iCycler Real-Time PCR Detection System (Bio-Rad). Quantification was achieved by the difference in quantification cycles (ΔΔCt) values that were normalized with RNA polymerase II as a reference control. Specificity of the qPCR primers were checked by melting curve analysis or gel electrophoresis of the qPCR product. The sequence of the primers used is listed in **Supplementary Table 1**.

### Chromatin Immunoprecipitation

Rat kidney (~40 mg) in small pieces was cross-linked in 1.5% formaldehyde (in PBS) at room temperature for 15 min. After adding glycine to a final concentration of 125 mM, kidney pellets were homogenized in Pierce™ IP lysis buffer (ThermoFisher Scientific) containing 1% (v/v) protease inhibitor cocktail and chromatin fragments of 500–1,000 base pair was obtained by sonication. The lysate was incubated with either 2 µg of specific primary antibody or IgG at 4°C overnight with rotation. Pierce™ Protein A Agarose beads (Thermo Fisher Scientific) were added to the lysates and incubated for another hour. After washing for four times, the chromatin fragments were eluted into a TE buffer containing proteinase K. qPCR was performed using 10% of the DNA samples and quantified by using the Ct values for normalization against the input control, for which 1% of the DNA samples were used for qPCR. Primers used are listed in **Supplementary Table 2**. Trimethyl-H3K4 (#17-614), H3K9 (#17-625), and H3K27 (#17-622) antibodies are purchased from Millipore.

### Protein Expression by Western Blotting

Kidney sample was homogenized in RIPA buffer by smashing in liquid nitrogen. Same amount of lysates protein (40 µg) was loaded and separated in *SDS-PAGE*. The resolved proteins were transferred onto nitrocellulose membranes and probed with specific primary antibody at 4°C overnight with agitation. GAPDH was probed as a loading control. The protein bands were visualized using enhanced chemiluminescence (ECL) reagents (GE Healthcare, Chicago, Illinois, United State) and developed in Fujitsu Biomedical film (Fujitsu, Japan). Densitometric analysis was performed using the ImageJ (NIH). Quantification protein expression was based on the ratio of target protein to GAPDH. The following primary antibodies were used: anti-ACE (Ab216476, Abcam, 1:500), anti-FGF2 (05-118, Millipore, 1:1000), anti-NHE3 (NB110-61586, Novus Biological, 1:2000), anti-Rac1 (Ab33186, Abcam, 1:1000), anti-Nr3c2 (Ab2774, Abcam, 1:1000, anti-Sgk1 (Ab59337, Abcam, 1:1000), anti-β-tubulin I (T7816, Sigma-Aldrich, 1:30000), and anti-GAPDH (2251-1, Epitomics, 1:30000).

### Chromatin Analysis

Chromatin accessibility was studied using the Micrococcal Nuclease (MNase, Cell Signaling Technology) digestion method. Kidney was homogenized and MNase digested for 1 h at 37°C. The genomic DNA was then isolated and collected using chromatin immunoprecipitation (ChIP) DNA purification kit (Active Motif). Quantitative PCR was performed using 5 ng DNA samples as template. In general, open chromatin regions were more susceptible to MNase digestion resulting in a greater delay of quantification cycle (C_T_), while closed chromatin regions were protected from the MNase digestion resulting in minimal delays in C_T_ value, compared to that of undigested templates. The sequence of the primers used is listed in **Supplementary Table 2**.

### Histological Staining

Frozen rat kidney was embedded in OCT medium and cryosectioning was performed to obtain slides with thickness of 7 µm. Trichrome Stain Kit (Abcam, Cambridge, UK) was used to analyze collagen fibers according to manufacturer’s instructions. In brief, the slides were incubated in the pre-heated Bouin’s Solution at 60°C for 1 h. The yellow color on the slides was removed by rinsing in running tap water followed by staining in Working Weigert’s Iron Hematoxylin Solution. The slides were then rinsed in deionized water and stained in Working Phosphomolybdic/Phosphotungstic Acid Solution followed by Aniline Blue Solution and 1% acetic acid. The slides were then rinsed, dehydrate and mounted. Collagen staining per glomeruli images was quantified by NIH ImageJ software.

### Blood Chemistry Detection

Blood samples were taken from the rats from each group and plasma was separated for the estimation of the various blood chemical parameters such as blood urea nitrogen (BUN) and potassium ion concentration. A blood chemical analyzer (Reflotron, Roche Co., Germany) was employed for this purpose using the specified analysis kits supplied from the manufacturer.

### Statistics

Results are expressed as mean ± SEM (standard error of the mean). Student’s t test was used for comparison of PETN group with control group. *P* values < 0.05 were considered significantly. GraphPad Prism (GraphPad Software, La Jolla, CA, USA) was used to generate graphs and statistical analysis.

## Results

### Maternal PETN Treatment Reduce Blood Pressure and Biomarkers of Kidney Injury in SHR

**Figure 1:**
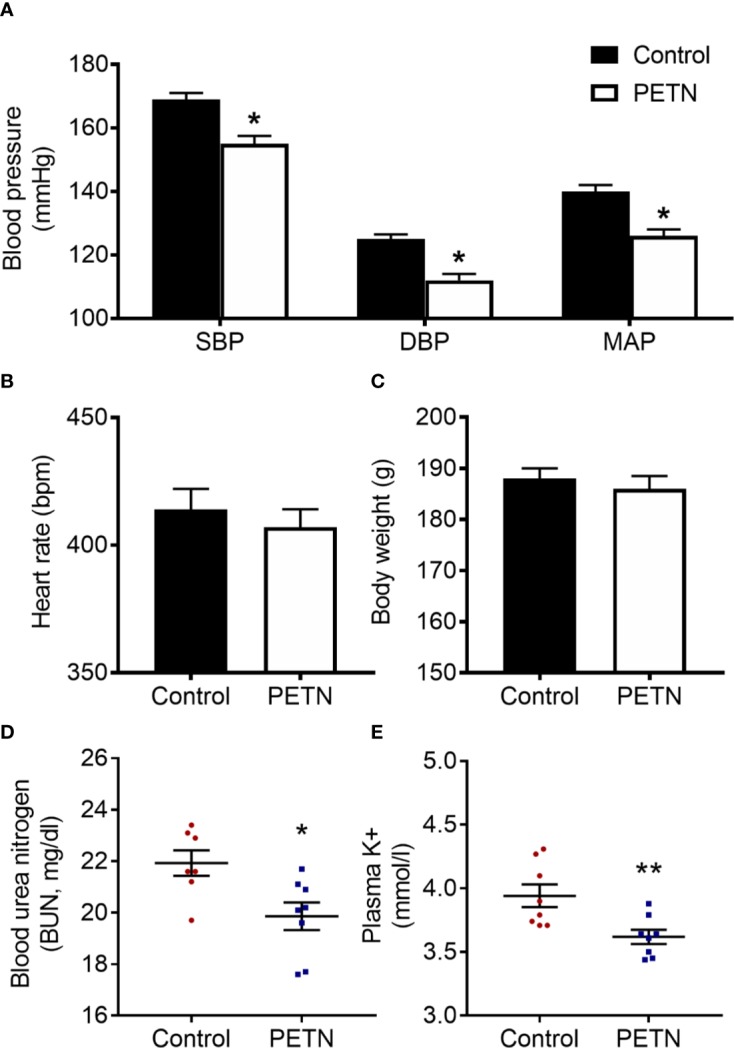
Maternal pentaerythritol tetranitrate (PETN) treatment leads to blood pressure and kidney injury reduction in early ages of female offspring (16 weeks). Parental spontaneously hypertensive rats (SHR) were treated with or without PETN (50 mg/kg/day) during pregnancy and lactation periods. Systolic blood pressure **(SBP)**, diastolic blood pressure **(DBP)**, and mean arterial pressure **(MAP) (A)**; heart rate **(B)** and body weight **(C)** were measured in the offspring at 16 weeks. Column represents mean ± SEM, n=25−26. Plasma samples were obtained from the female offspring (16 weeks) from each group for the measurement of the blood urea nitrogen **(BUN) (D)** and potassium ion concentration **(E)** using a blood chemical analyser (Reflotron, Roche Co., Germany). Student’s t test was used for comparison of PETN group with control group. *P < 0.05; **P < 0.01; vs. control group.

In F1 offspring, we measured the blood pressure at the age of 16 weeks using the tall-cuff method. The systolic, diastolic and mean blood pressure of PETN group was significantly lower compared to control (**Figure 1A**). The mean arterial blood pressure of PETN group was ~15 mmHg lower than that of control group (**Figure 1A**). No significant difference in heart rate or body weight was found in the F1 female offspring at 16-weeks old (**Figures 1B, C**). This is consistent with our previous study showing blood pressure reduction at the age of 6–8 months only in female offspring. Therefore, the following experiments were only performed with kidneys from 16-week-old female offspring. The plasma BUN and K^+^ concentration of the F1 female offspring in PETN group were significantly reduced compared to control (**Figures 1D, E**).

### Maternal PETN Treatment Leads to Differential Gene Expression in F1 SHR Kidney

**Figure 2:**
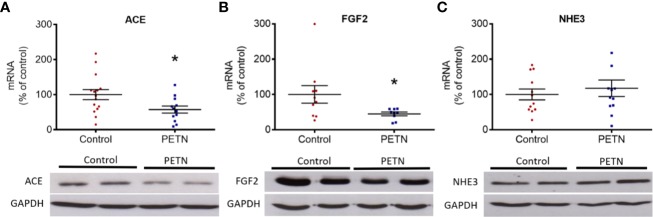
Maternal pentaerythritol tetranitrate (PETN) treatment leads to differential gene expression in the kidney of female offspring. Parental spontaneously hypertensive rats (SHR) were treated with or without PETN (50 mg/kg/day) during pregnancy and lactation periods. The gene and protein expression of angiotensin-converting enzyme (ACE) **(A)**, basic fibroblast growth factor (FGF2) **(B)**, and sodium-hydrogen antiporter 3 (NHE3) **(C)** were studied with quantitative real-time PCR and Western blotting using kidney of 16-week-old offspring, respectively. RNA polymerase II was used as reference gene for quantitative PCR (qPCR) and GAPDH was used as internal control for the protein loading in Western blotting. Column represents mean ± SEM, n=8–12. Student’s t test was used for comparison of PETN group with control group. *P < 0.05, vs. control group.

We studied the mRNA expression of genes of the angiotensin system and of those involved in ion transportation, nephrogenesis, fibrosis, or apoptosis. No changes were found in the mRNA expression of angiotensin II receptors (Agtr1a, Agtr1b, and Agtr2) or ACE2 (data not shown). Interestingly, ACE (ACE1) expression was found lower in the kidney of PETN offspring (**Figure 2A**). We analyzed the mRNA expression of odd-skipped-related 1 (OSR1), glial cell-derived neurotrophic factor (Gdnf), GATA binding protein 3 (Gata3), and leukemia inhibitory factor (Lif). No differences were found in the expression of these genes involved in nephrogenesis between PETN and control offspring (data not shown). The renal expression of FGF2, a key factor driving kidney fibrosis, was found decreased by maternal PETN treatment, both at mRNA and protein levels (**Figure 2B**). Among the membrane transport proteins, we found no significant changes in the mRNA expression of Na^+^-K^+^-Cl^-^ cotransporters (NKCC1 and NKCC2), Na^+^-Cl^−^ cotransporter (NCC), the sodium/potassium-transporting ATPase subunit alpha-1 (ATP1a1) or the sodium-hydrogen exchanger 2 (NHE2) (data shown exemplarily for NHE3 in **Figure 2C**). No effect on the expression of genes involved in apoptosis and cell survival (data not shown).

### Maternal PETN Treatment Induces Epigenetic Changes in F1 SHR Kidney

**Figure 3:**
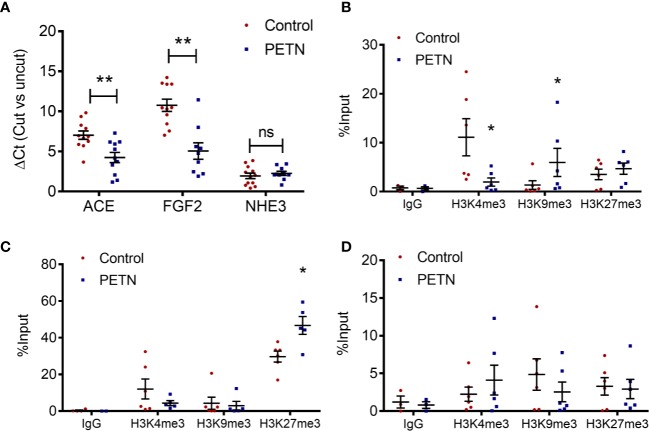
Maternal pentaerythritol tetranitrate (PETN) treatment leads to epigenetic changes in the kidney of female offspring. Parental spontaneously hypertensive rats (SHR) were treated with or without PETN (50 mg/kg/day) during pregnancy and lactation periods. **(A)** The chromatin accessibility at the proximal promotor regions around the transcription start site of ACE, FGF2, and NHE3 was studied by MNase digestion. Histone 3 lysine 4 trimethylation (H3K4me3), histone 3 lysine 9 trimethylation (H3K9me3), and histone 3 lysine 27 trimethylation (H3K27me3) at the proximal promotor regions around the transcription start site of angiotensin-converting enzyme (ACE) **(B)**, fibroblast growth factor (FGF2) **(C)** and sodium-hydrogen antiporter 3 (NHE3) **(D)** were studied with chromatin immunoprecipitation (ChIP) followed by quantitative PCR using kidney of 16-week-old offspring. Nonspecific IgG was used as negative control of the ChIP experiment. Column represents mean ± SEM, n= 6. Student’s t test was used for comparison of PETN group with control group. Student’s t test was used for comparison of PETN group with control group. *P < 0.05; **P < 0.01 vs. control group.

The accessibility and the histone modification in the proximal promoter regions at the transcriptional start site of ACE, FGF2, and NHE3 were examined. MNase digestion experiment shows a significant reduction in the ΔCt of ACE and FGF2 in PETN group compared to control, whereas that of NHE3 had no significant difference (**Figure 3A**). This indicates a reduction of chromatin accessibility in the proximal promoter regions of ACE and FGF2 gene in the kidney of PETN group.

In the proximal promoter regions around the transcription start site of ACE, H3K4 trimethylation was significantly reduced and H3K9 trimethylation was enhanced in the PETN group (**Figure 3B**). In the proximal promoter regions around the transcription start site of FGF2, H3K27 trimethylation was significantly enhanced in the PETN group (**Figure 3C**). In line with the MNase digestion result, H3K4, H3K9, and H3K27 trimethylation show no significant changes in the proximal promoter regions around the transcription start site of NHE3 (**Figure 3D**). H3K9 and H3K27 trimethylation are repressive epigenetic marks associated with transcriptional repression while H3K4 trimethylation are epigenetic marks in transcriptional active euchromatin and associated with transcriptional activation. The above results indicate reduced ACE and FGF2 gene expression by epigenetic control in the kidney of PETN group.

### Maternal PETN Treatment Reduces Kidney Fibrosis in Young SHR

**Figure 4:**
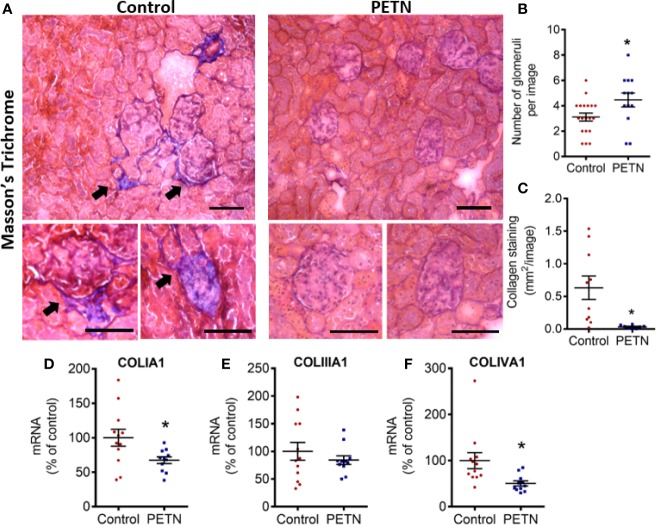
Maternal pentaerythritol tetranitrate (PETN) treatment reduces kidney fibrosis in the female offspring. Parental spontaneously hypertensive rats (SHR) were treated with or without PETN (50 mg/kg/day) during pregnancy and lactation periods. **(A)** The collagen deposition in the kidney of the 16-week-old female offspring was examined by Masson’s Trichrome staining. Blue staining pointed by arrow represents collagen. Representative images were shown. Scale bar, 100 µm. **(B)** Number of glomeruli was counted. Bar chart represents average counts from 10 images. **(C)** Collagen deposition around glomeruli was quantified per image. Bar chart represents average value from 10 images. The gene expression of collagen type 1 **(D)**, type III **(E)**, and type IV **(F)** in the kidney of 16-week-old offspring were studied with quantitative real-time PCR. RNA polymerase II was used as reference gene for qPCR. Column represents mean ± SEM. n=11−12. Student’s t test was used for comparison of PETN group with control group. *P < 0.05, vs. control group.

**Figure 5:**
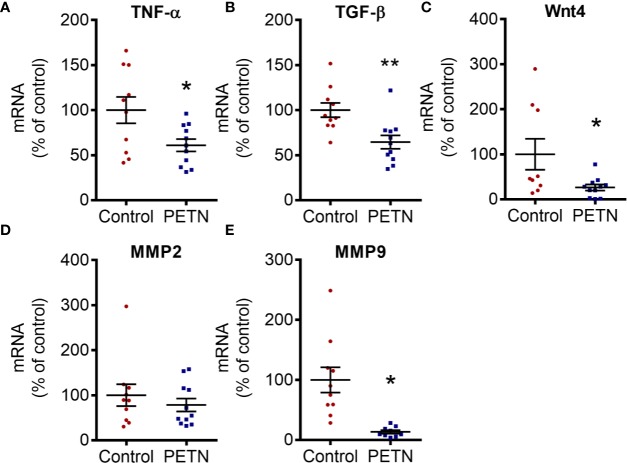
Maternal pentaerythritol tetranitrate (PETN) treatment downregulates profibrotic signaling pathways in the female offspring. Parental spontaneously hypertensive rats (SHR) were treated with or without PETN (50 mg/kg/day) during pregnancy and lactation periods. The mRNA expression of tumor necrosis factor (TNF-α) **(A)**, Wnt4 **(B)**, transforming growth factor beta (TGF-β) **(C)** and matrix metalloproteinase (MMP2 and MMP9) **(D**, **E)** in the kidney of 16-week-old offspring were studied with quantitative real-time PCR. RNA polymerase II was used as reference gene for qPCR. Column represents mean ± SEM, n=10–11. Student’s t test was used for comparison of PETN group with control group. *P < 0.05, **P < 0.01; vs. control group.

In order to examine renal fibrosis and collagen deposition, kidney samples were fixed and stained with Masson’s trichrome. Masson’s trichrome staining showed that the blue stain (indicating collagen deposition) was significantly reduced in the kidney of the PETN group compared to that of control. The collagen deposition in the kidney of control SHR was found mainly around the glomeruli (**Figure 4A**). PETN group showed a significant increase in the number of glomeruli in the kidney (**Figure 4B**), and significant reduction in collagen staining compared to that of control (**Figure 4C**). Moreover, qPCR results showed that collagen type I a1 (COLIA1) and type 4 a1 (COLIVA1) was downregulated in the kidney of PETN group (**Figures 4D–F**). Furthermore, fibrosis-related signaling was examined by measuring the gene expression of Wnt4, tumor necrosis factor (TNF-α), matrix metalloproteinases (MMP2 and MMP9), and transforming growth factor beta (TGF-β) in the kidney. The mRNA expressions of the Wnt4, TNF-α, and TGF-β were significantly downregulated in PETN group (**Figures 5A–C**). MMP2 gene expression in the kidney of PETN group was not changed whereas MMP9 expression was significantly reduced by maternal PETN treatment (**Figures 5D, E**). These results indicate that the reduction of sclerotic glomeruli in the kidney of the PETN group is likely to be mediated by TNF-α and Wnt4 signaling.

### Maternal PETN Treatment Prevent Activation of Mineralocorticoid Receptor in the Kidney

**Figure 6:**
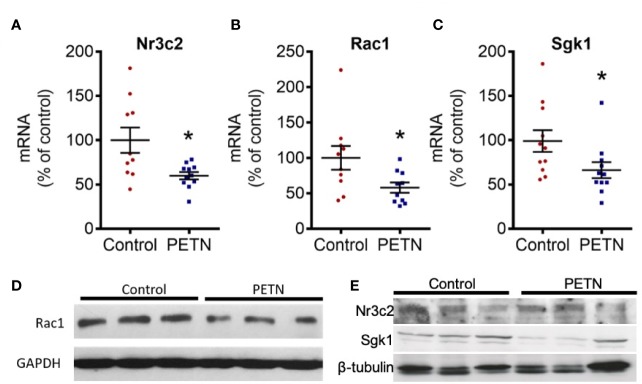
Maternal pentaerythritol tetranitrate (PETN) treatment prevents activation of mineralocorticoid receptor in the kidney female offspring. Parental spontaneously hypertensive rats (SHR) were treated with or without PETN (50mg/kg/day) during pregnancy and lactation periods. The gene expression of mineralocorticoid receptor (MR or Nr3c2) **(A)**, Ras-related C3 botulinum toxin substrate 1 (Rac1) **(B)** and serum and glucocorticoid-regulated kinase 1 (Sgk1) **(C)** in the kidney of 16-weeks old offspring were studied with quantitative real-time PCR. RNA polymerase II was used as reference gene for qPCR. Protein expressions Rac1 **(D)** Nr3c2 and Sgk1 **(E)** were analyzed by Western blotting using kidney of 16-week-old offspring. GAPDH or β-tubulin was used as internal control for the protein loading in Western blotting. Column represents mean ± SEM, n=10–11. Student’s t test was used for comparison of PETN group with control group. *P < 0.05, vs. control group.

In kidney, aldosterone mediates salt and water homeostasis by binding to the mineralocorticoid receptor (MR or Nr3c2). Activation of MR by aldosterone contributes to kidney damage in experimental models of hypertension (Blasi et al., 2003). Therefore, we examined the gene expression of MR-signaling pathway including Ras-related C3 botulinum toxin substrate 1 (Rac1) and glucocorticoid-regulated kinase 1 (Sgk1). The mRNA expression of MR, Rac1, and Sgk1 in the kidney of PETN group was significantly reduced compared to control (**Figures 6A–C**). The protein expression of Rac1 and Sgk1 in the kidney of PETN group was also significantly reduced compared to control (**Figures 6D, E**). These results indicate a downregulation of the aldosterone-MR signaling pathway in the kidney of PETN group.

### Maternal PETN Treatment Leads to Reduction in Macrophage Infiltration and Inflammation in Kidney of Female Offspring

**Figure 7:**
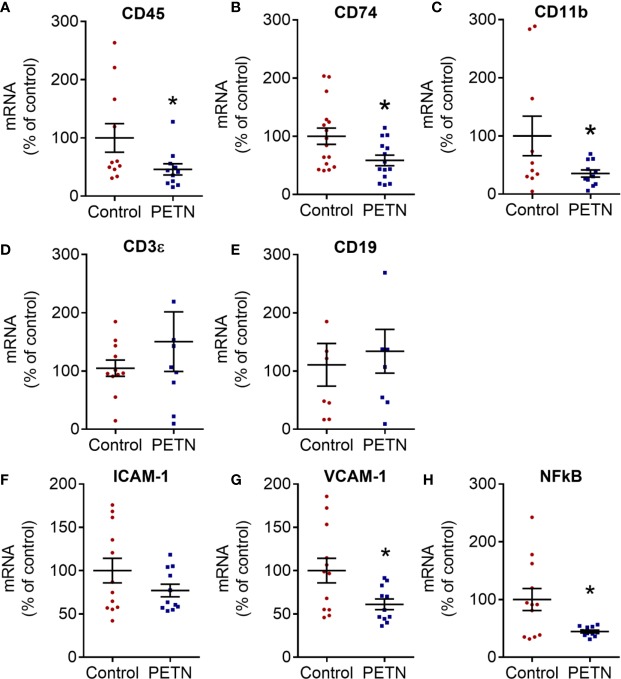
Maternal pentaerythritol tetranitrate (PETN) treatment leads to reduction in macrophage infiltration in kidney of female offspring. Parental spontaneously hypertensive rats (SHR) were treated with or without PETN (50 mg/kg/day) during pregnancy and lactation periods. Different cell surface markers of antigen presenting cells were measured by the gene expression of cluster of differentiation (CD) 74 **(A)**, CD 45 **(B)**, CD 11B **(C)**, CD3ϵ **(D)**, and CE19 **(E)** in the kidney of 16-week-old female offspring. Proinflammatory gene expression of intercellular adhesion molecule-1 (ICAM-1) **(F)**, vascular cell adhesion protein 1 (VCAM-1) **(G)** and nuclear factor kappa-light-chain-enhancer of activated B cells p105 subunit (NF-κB) **(H)** in the kidney of 16-week-old female offspring were measured. RNA polymerase II was used as reference gene for qPCR. Column represents mean ± SEM, n=8−12. Student’s t test was used for comparison of PETN group with control group. *P < 0.05, vs. control group.

Immune cell infiltration in the kidney was analyzed by the gene expression of several cell surface markers of leukocytes. The CD45, CD74 and CD11b gene expression was significantly reduced in the PETN group compared to control (**Figures 7A–C**). There is not significant difference in the mRNA expression of CD3ϵ and CD19 (**Figures 7D, E**). CD45 is a leukocyte common antigen, CD74 is expressed in macrophages, CD 11b is a pan-macrophage marker, whereas CD3ϵ is expressed during T-cell development and CD19 is a B-lymphocyte antigen. Therefore, the results suggest a reduction of the numbers of macrophages but not T- or B- lymphocytes in the kidney of PETN offspring.

The gene expression of intercellular adhesion molecule-1 (ICAM-1) showed a trend of reduction in the PETN group (**Figure 7F**) while vascular cell adhesion molecule 1 (VCAM-1) and NF-κB was significantly reduced in the PETN group compared to control (**Figures 7G, H**). The results indicate a reduction of inflammation in the kidney of PETN group.

## Discussion

In this study, we show that maternal treatment of SHR with PETN during the pregnancy and lactation periods leads to a reduction in blood pressure at the age of 16 weeks. This was associated with a reduction of kidney fibrosis, MR signaling and kidney macrophage infiltration and inflammation. Maternal PETN treatment reduced the renal expression of ACE and FGF2 *via* epigenetic changes (histone modifications). These renal effects may represent molecular mechanisms contributing to the reduction of blood pressure in the PETN offspring.

According to international REPROTOX database (http://reprotox.org), PETN is safe for the usage in pregnancy with no obvious risk of congenital anomalies. There is no reproductive or developmental toxicity observed so far in rats during mating and pregnancy period (Quinn et al., 2009). In our previous study (Wu et al., 2015), we demonstrated that maternal treatment of PETN in SHR during the pregnancy and lactation periods led to a persistent reduction of blood pressure in 8-month-old female offspring, with improved endothelial functions *via* enhancing NO production. These data shed light on the blood pressure reprogramming effects by perinatal PETN treatment. However, the development of hypertension is not solely the results of vascular dysfunction. Therefore, the current study was designed to investigate the kidney of PETN offspring as kidney is also an important organ for fluid and blood pressure homeostasis.

Our studies show that maternal treatment of SHR with PETN during the pregnancy and lactation periods effectively lowers blood pressure in the female offspring starting from 16 weeks and this effect persists at least to the age of 8 months. Maternal PETN treatment had no effects on bloods pressure in the male offspring at the age of 16 weeks (data not shown). Endothelial dysfunction was found only in SHRs older than 6 months; but not in younger SHRs (Bernatova et al., 2009). The development of renal damage is crucial in the pathogenesis of hypertension in SHR with renal salt retention, inflammation, and fibrosis being the major contributors to the hypertensive phenotype in SHR (Roman, 1987; Evan et al., 1990; Hultström, 2012). Our results suggest a possible improvement of kidney function in the 16-weeks old F1 PETN female offspring. Therefore, we investigated the renal effects of maternal PETN treatment in younger offspring (4 months of age).

PETN exerts the beneficial effect in the kidney of young SHR by reducing the expression of ACE and FGF2 *via* epigenetic mechanisms. Inhibition of ACE attenuates the decline in renal function and structural damages in kidney diseases (Anderson et al., 1985; Rosenberg et al., 1994). Aldosterone stimulates the renal salt reabsorptions *via* MR-induced Sgk1 expression; whereas elevated MR-Sgk1 signaling was associated with the pathogenesis of kidney fibrosis and hypertension (Lang et al., 2000; Terada et al., 2005; Kawarazaki et al., 2012). Our results suggest that maternal PETN treatment effectively prevents the activation of the MR-Sgk1 signaling pathway in SHR kidney. The epigenetic downregulation of FGF2 by PETN maternal treatment also contributes to the reduction of renal fibrogenesis in the PETN offspring. FGF2 is an important cytokine which facilitates renal matrix formation (Strutz, 2009). FGF2 is highly associated with several kidney pathologies in both animal models and patients with chronic kidney disease (Strutz et al., 2001; Mattison et al., 2012; Bozic et al., 2018). Although the direct causal relation between FGF2 and renal fibrosis has not been demonstrated yet, FGF2 is associated with TGFβ-induced proliferation of renal fibroblast, together with the induction of other profibrotic signaling molecules including Wnt4 and TNF-α (Anderson et al., 1985; Strutz et al., 2001). In our model, renal fibrosis in the SHR mice is significantly reduced in the PETN group evidenced by the reduction in collagen deposition in the kidney glomerular and the gene expression in fibrosis-related signaling pathways (Yamamoto et al., 1994; Surendran et al., 2002; Tan et al., 2014). Collagen I is usually upregulated in fibrotic glomerular while our result suggested that collagen I expression can be downregulated by maternal PETN treatment (Stokes et al., 2001). Maternal PETN treatment leads to a significant reduction in expression of major profibrotic cytokines Wnt4, TNF-α, and TGF-β in SHR kidney. Reduced collagen deposition and sclerotic glomeruli in the kidney of PETN group was associated with the downregulation of renal MMP9. On the other hand, there is evidence showing that renal inflammation is already provoked at the stage of prehypertension with augmented macrophage infiltration (Harrison et al., 2011). Prolonged inflammation could lead to fibrosis and kidney failure (Guiteras et al., 2016). Our data also shows that maternal PETN treatment significantly reduced immune cell infiltration and renal inflammation in SHR offspring.

**Figure 8:**
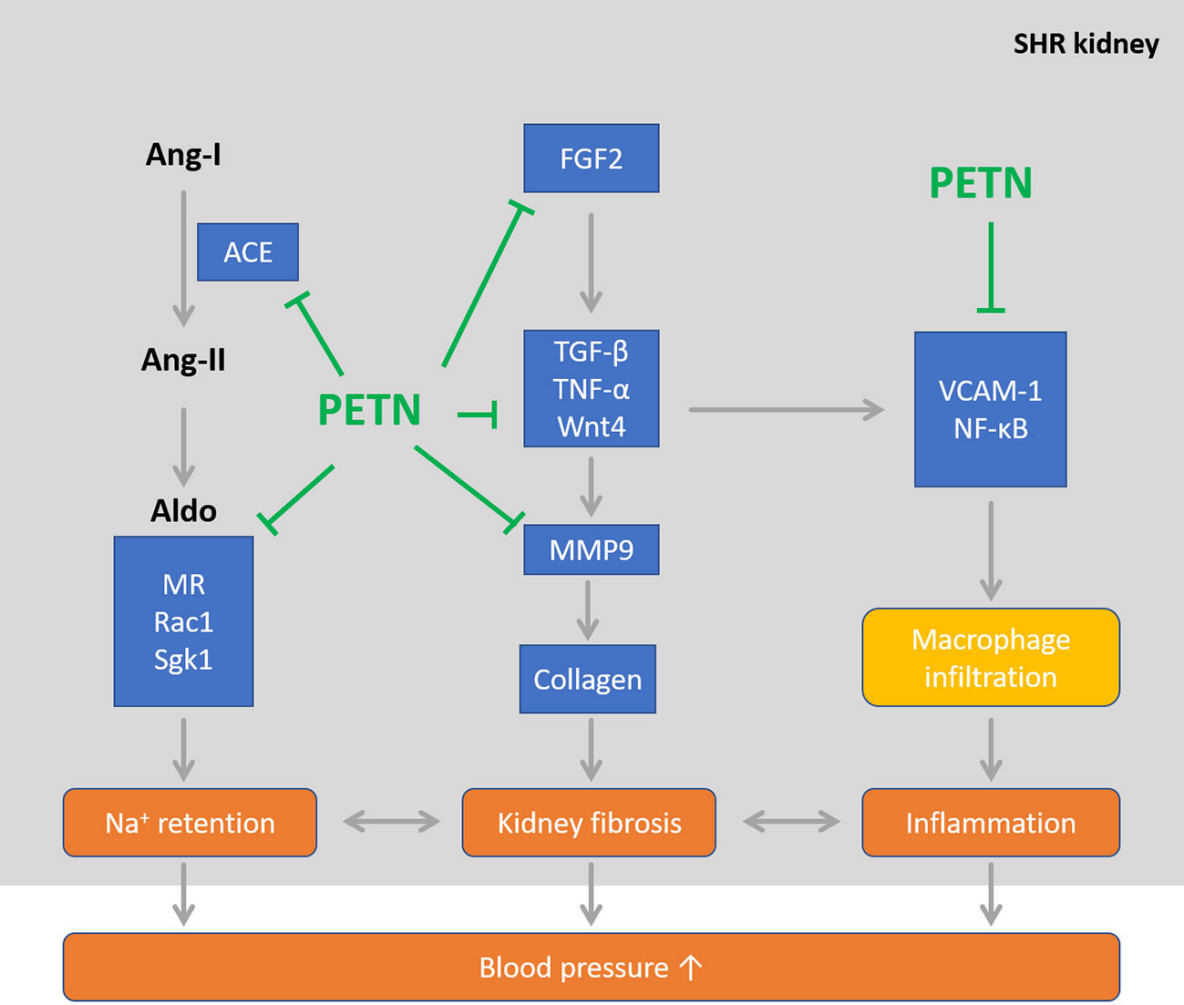
Maternal pentaerythritol tetranitrate (PETN) treatment persistently reduces blood pressure in spontaneously hypertensive rats (SHR) offspring *via* improving kidney phenotype in young age. PETN treatment could be important in improving the renal-cardiovascular system by its effect in fetal reprogramming *via* epigenetics modifications in early remodeling of kidney.

Our results suggest that maternal PETN treatment leads to epigenetic modifications which results in long-lasting changes in gene expression. The downregulation in various signaling pathways including Wnt4 and angiotensin-aldosterone, results in the reduction in renal inflammation and kidney fibrosis events in the young SHR offspring. We believe that this early remodeling of the young SHR kidney by maternal PETN treatment during pregnancy and lactation periods is critical in persistently maintain the lower blood pressure in the offspring. While vascular dysfunction occurs later in older mice, PETN treatment also improves vascular function *via* enhancing NO production. Together, these mechanisms exerted by the maternal PETN treatment persistently protect the offspring from hypertension. Therefore, a hypothesis for the underlying mechanism of maternal PETN treatment *via* early kidney remodeling in preventing hypertension persistently is proposed (**Figure 8**). Our results suggest that PETN treatment could be important in improving the renal-cardiovascular system by its effect in fetal reprogramming *via* epigenetics modifications. However, more research is needed to investigate the long-term effects and potential adverse effects of maternal PETN treatment.

Molecular mechanisms how PETN leads to epigenetic changes are out of the scope of the present study. PETN is an organic nitrate currently in clinical use for angina pectoris. Among the organic nitrates, PETN is the only drug devoid of nitrate tolerance and endothelial dysfunction (Münzel et al., 2005; Wenzel et al., 2007). PETN not only releases NO like other organic nitrates, but also additionally enhances endothelial NO production by preventing eNOS uncoupling (Schuhmacher et al., 2010; Knorr et al., 2011; Schuhmacher et al., 2011). Moreover, PETN also possesses multiple antioxidative effects including induction of heme oxygenase 1 (HO-1) (Schuhmacher et al., 2010) and extracellular superoxide dismutase (SOD3) (Oppermann et al., 2009; Schuhmacher et al., 2011), inhibition of cardiac NADPH oxidase activity and serum xanthine oxidase activity (Schuhmacher et al., 2011). Therefore, the epigenetic effects of PETN may be attributable to the enhanced NO production and reduced oxidative stress. NO plays an important role in regulating the uteroplacental perfusion (Kulandavelu et al., 2012) and the fetoplacental circulation (Kulandavelu et al., 2013), which may lead to changes of in-utero environment. Recently, NO has been identified as an epigenetic regulator by three distinct mechanisms: direct inhibition of the JmjC class lysine-specific histone demethylases (KDM), reduction in iron cofactor availability, and expressional regulation of KDM and lysine methyltransferases (KMTs) (Hickok et al., 2013). Treatment of cells with physiological NO concentrations results in changes of histone methylation patterns that significantly influence chromatin structure and gene transcription (Hickok et al., 2013). In addition, NO also enhances the expression of sirtuin 1 (SIRT1), a member of the class III, NAD^+^-dependent histone deacetylases (Ota et al., 2008). Oxidative stress, on the other hand, may lead to epigenetic dysregulation through a large range of mechanisms (Cyr and Domann, 2011). For instance, reactive oxygen species (ROS) directly inhibits HDAC activity by inducing tyrosine nitration on the HDAC enzymes (Cyr and Domann, 2011). Change in critical redox intermediates [such as NAD^+^, S-adenosyl methionine (SAM), and 2-oxoglutarate] modulates pathways that alter numerous epigenetic marks, including histone methylation, acetylation, as well as DNA methylation (Cyr and Domann, 2011). SHR have elevated levels of oxidative stress (Li et al., 2006). Therefore, reduction in oxidative stress by PETN may modulate the redox-sensitive pathways of epigenetic regulation. Collectively, we hypothesize that the combined effects (i.e. enhancement of NO bioavailability and reduction of oxidative stress) represent the major molecular mechanisms underlying the epigenetic modifications induced by PETN maternal treatment.

In conclusion, our studies show that maternal PETN treatment of rat with genetic hypertension leads to persistent blood pressure reduction in the female offspring. This effect can be observed from the age of 4 months and lasts at least to the age of 8 months. In addition to the improvement of endothelial dysfunction reported previously, the present study demonstrates that maternal PETN treatment also reduces MR signalling and prevents kidney inflammation and fibrosis.

## Translational Perspective

PETN is an organic nitrate devoid of nitrate tolerance and shows no reproductive or developmental toxicity in animal studies. We have previously shown that maternal PETN treatment of hypertensive rats persistently reduced blood pressure and improved endothelial function resulting from epigenetic changes in the female offspring. In the present study, we demonstrate that maternal PETN treatment of hypertensive rats significantly reduces aldosterone signaling and ameliorates inflammation and fibrosis in the kidney of female offspring. Thus, maternal treatment may represent a promising epigenetic therapy preventing hypertension and kidney diseases in the next generation. This is especially of therapeutic interest for high-risk patients, i.e., those with family history of severe hypertension, preeclampsia or abnormal placental function.

## Data Availability Statement

The datasets used in the current study are available from the corresponding authors on reasonable request.

## Ethics Statement

The animal experiment was approved by the responsible regulatory authority (Landesuntersuchungsamt Rheinland-Pfalz; 23 177-07/G 11-1-015) and was conducted in accordance with the German animal protection law and the National Institutes of Health (NIH) Guide for the Care and Use of Laboratory Animals.

## Author Contributions

NX and HL designed the study. AM, MC, ZW, and GR performed the experiments and analyzed data. AM wrote the manuscript. AD, TM, NX, and HL critically reviewed and edited the manuscript. All authors agreed to its publication.

## Funding

This work was supported by the Deutsche Forschungsgemeinschaft [DFG, grant LI-1042/3-1], by the Center for Translational Vascular Biology (CTVB) and the Center for Thrombosis and Hemostasis (CTH, funded by the Federal Ministry of Education and Research, BMBF 01EO1003) of Johannes Gutenberg University Medical Center, Mainz, Germany. TM is PI of the DZHK (German Center for Cardiovascular Research), Partner Site Rhine-Main, Mainz, Germany. MC and ZW were supported by China Scholarship Council.

## Conflict of Interest

The authors declare that the research was conducted in the absence of any commercial or financial relationships that could be construed as a potential conflict of interest.

PETN used in this study was provided by Actavis Deutschland (now PUREN Pharma GmbH & Co. KG, Munich, Germany).
