# Supplementary material for: Renal Effects of Fetal Reprogramming With Pentaerythritol Tetranitrate in Spontaneously Hypertensive Rats
Source: Front Pharmacol. 2020 Apr 29;11:454. doi: 10.3389/fphar.2020.00454 (PMC7201020; doi:10.3389/fphar.2020.00454)
Supplement: Supplementary file 1 [file DataSheet_1.pdf]

Renal effects of fetal reprogramming with pentaerythritol tetranitrate in spontaneously hypertensive rats

Andy W C Man[1†] ,Min Chen[1,2†], Zhixiong Wu[1], Gisela Reifenberg[1], Andreas Daiber[3,4], Thomas Münzel[3,4], Ning Xia[1], Huige Li[1*]

[1] Department of Pharmacology, Johannes Gutenberg University Medical Center, 55131 Mainz, Germany; [2] Department of Anaesthesiology, Institute of Anaesthesiology and Critical Care Medicine, Union Hospital, Tongji Medical College, Huazhong University of Science and Technology, Wuhan, China. [3] Center for Cardiology, Cardiology I - Laboratory of Molecular Cardiology, Johannes Gutenberg, University Medical Center, Mainz, Germany; [4] German Center for Cardiovascular Research (DZHK), Partner Site Rhine-Main, Mainz, Germany.

Correspondence:
Dr. Ning Xia or Prof. Dr. Huige Li, Department of Pharmacology, Johannes Gutenberg University Medical Center, Obere Zahlbacher Str. 67, 55131 Mainz, Germany. E-Mail: xianing@uni-mainz.de (N.X.); huigeli@uni-mainz.de (H.L.)

[†] These authors contributed equally to this work and should be both considered as first author.

Running title: fetal reprogramming in kidney

Original article

Key words: fetal programming; epigenetics; spontaneously hypertensive rats; pentaerythritol tetranitrate; kidney fibrosis

Supplementary Files

Supplementary Tables.

Supplementary Table 1. Primer list for gene expression studies using qPCR

ACE –F	CAGAGGCCAACTGGCATTAT
ACE -R	CTGGAAGTTGCTCACGTCAA
FGF2 -F	TCCATCAAGGGAGTGTGTGC
FGF2 -R	TCCGTGACCGGTAAGTGTTG
NHE3 -F	AACTCTCCCAGCAACTTCCG
NHE3 -R	GCCTGTATCACATGTGTGTGGA
TNFα -F	ATGGGCTCCCTCTCATCAGT
TNFα -R	GCTTGGTGGTTTGCTACGAC
Wnt4 -F	AGGTGGTGACACAAGGGACC
Wnt4 -R	CCATAGGCGATGTTGTCCGA
MMP2 -F	CAAGCCCAAGTGGGACAAGA
MMP2 -R	CCATGCTCCCATCGACCAAA
MMP9 -F	AGCCGACGTCACTGTAACTG
MMP9 -R	AACAGGCTGTACCCTTGGTC
TGFβ -F	CTGCTGACCCCCACTGATAC
TGFβ -R	AGCCCTGTATTCCGTCTCCT
Nr3c2 -F	AATAACGTCCCTCTGCGCTC
Nr3c2 -R	GCCTGAAGTGGCATAGCTGA
Rac1 -F	CAATGCGTTCCCTGGAGAGT
Rac1 -R	AACACGTCTGTTTGCGGGTA
Sgk1 -F	TAGCCTCACCATCCCGTTTG
Sgk1 -R	CATCTGCGTACCCGCTTCTT
CD74 -F	TCCCTGGACACAACAAATGA
CD74 -R	AAATGGGGGTCCCTTAGATG
CD45 -F	AGGAAACTTGCTCCCCATCC
CD45 -R	TGTTCCTTGCCCTGTGACAA
CD11b -F	GGCCTGTACAAGCTTGGCTTT
CD11b -R	GCTGTTGGTCTGACCGTCTA
CD3e -F	AGTAATGAGCCAGCCGTGTC
CD3e -R	AGACTTCATACTCTTCCTGGCA
CD19 -F	CACCCATGGTTCATTGCCCA
CD19 -R	GCACAACATTGTCTCCCTCTTC
ICAM -F	CGGGAGATGAATGGTACCTACAA
ICAM -R	TGCACGTCCCTGGTGATACTC
VCAM -F	GAAGCCGGTCATGGTCAAGT
VCAM -R	GGTCACCCTTGAACAGTTCTATCTC
NFκB1-F	GCTTACGGTGGGATTGCATT
NFκB1-R	TTATGGTGCCATGGGTGATG
Pol II-F	AACGAACTGCCCGGCTTAC
Pol II-R	CCCTTCAACCGTTGCTTCAC

Supplementary Table 2. Primer list for chromatin accessibility and ChIP-qPCR studies

Primer	Primer sequence	Target Gene ID
FGF2 -F	GCGGTTTTCATCTTCCCACG	54250
FGF2 -R	GAGACAGAGGCCAGGAATGG	
NHE3 -F	GGTGGCCTTTAATCCCGCTG	24784
NHE3 -R	ACTGTTTGCATTCACGTGCG	
ACE –F	TTTATAATCCGCAGGGCGGT	24310
ACE -R	CCCATGGCGCGGTGC	
